# Synthesis, characterization, Hirshfeld surface analysis, antioxidant and selective *β*-glucuronidase inhibitory studies of transition metal complexes of hydrazide based Schiff base ligand

**DOI:** 10.1038/s41598-023-49893-6

**Published:** 2024-01-04

**Authors:** Farzia, Sadia Rehman, Muhammad Ikram, Adnan Khan, Rizwan Khan, Mutasem Omar Sinnokrot, Momin Khan, Abdullah F. AlAsmari, Fawaz Alasmari, Metab Alharbi

**Affiliations:** 1 Department of Chemistry, Abdul Wali Khan University, Mardan, Pakistan; 2 School of Physics & the Key Laboratory of Weak Light Nonlinear Photonics, Ministry of Education, Nankai University, Tianjin, 300071 People’s Republic of China; 3 Department of Zoology, Abdul Wali Khan University, Mardan, Pakistan; 4 College of Arts and Sciences, American University of Iraq-Baghdad, Airport Road Baghdad, Baghdad, Iraq; 5 Department of Pharmacology and Toxicology, College of Pharmacy, King Saud University, 11451 Riyadh, Saudi Arabia

**Keywords:** Coordination chemistry, Inorganic chemistry

## Abstract

The synthesis of N′-[(4-hydroxy-3-methoxyphenyl)methylidene] 2-aminobenzohydrazide (H-AHMB) was performed by condensing O-vanillin with 2-aminobenzohydrazide and was characterized by FTIR, high resolution ESI(+) mass spectral analysis, ^1^H and ^13^C-NMR. The compound H-AHMB was crystallized in orthorhombic *Pbca* space group and studied for single crystal diffraction analysis. Hirshfeld surface analysis was also carried out for identifying short interatomic interactions. The major interactions H…H, O…H and C…H cover the Hirshfeld surface of H-AHMB. The metal complexes [M(AHMB)n] where M = Co(II), Ni(II), Cu(II) and Zn(II) were prepared from metal chlorides and H-AHMB ligand. The bonding was unambigously assigned using FTIR and UV/vis analysis. The synthesized ligand H-AHMB and its metal complexes were studied for *β*-glucuronidase enzyme inhibition. Surprisingly the metal complexes were found more active than the parent ligand and even the standard drug. Zn-AHMB shown IC_50_ = 17.3 ± 0.68 µM compared to IC_50_ = 45.75 ± 2.16 µM shown by d-saccharic acid-1,4-lactone used as standard. The better activity by Zn-AHMB implying zinc based metallodrug for the treatment of diseases associated with *β*-glucuronidase enzyme. The DPPH radical scavenging activities were also studied for all the synthesized compounds. The Co-AHMB complex with IC_50_ = 98.2 ± 1.78 µM was the only candidate to scavenge the DPPH free radicals.

## Introduction

**Scheme 1:**
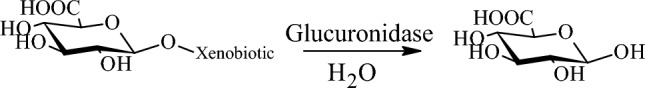
Conversion of *β*-d-glucuronides to *β*-d-glucuronic acid.

Molecular target therapy is the most popular approach in the field of medicinal chemistry to design drugs with improved therapeutic effects. Due to their importance in numerous biological processes, glycosyl hydrolases (GHs) are one of these targets. GHs inhibitors are involved in antiviral, anticancer and antidiabetic drug development^1–3^. A member of the group of GHs, *β*-glucuronidase (GLU) is an enzyme that is present in the endoplasmic reticulum, bodily fluids, and mammalian lysosomes^4^. It has also been observed in plants, fishes, insects and mollusks and catalyzes the conversion of *β*-d-glucuronides to their corresponding aglycone and *β*-d-glucuronic acid through hydrolysis as shown in Scheme 1.

*β*-Glucuronides enzyme is overly expressed in various types of cancers like breast^5^, cervical^6^, colon^7^, lung^8^, renal carcinoma and leukemia^9^. It has been found that *β*-GLU also effect other organs when overly expressed causing certain infections like urinary tract infection^10^, diabetes^11^, neuropathy^12^, HIV^12^ and rheumatoid arthritis^13^.* β*-glucuronidase enzyme has been shown to be released abnormally in chronic rheumatoid arthritis (RA). As a result of the RA's impact on the blood vessels, heart, lungs, muscles, and joints, bone deformities and osteoporosis develops^14^.

Therefore, inhibition of *β*-glucuronidase enzyme is deemed as potential molecular target for designing anticancer, anti-inflammatory, antidiabetic and antineuropathic drug^15–20^. Plethora of potential inhibitors of *β*-Glucuronides exists comprising of both organic and coordination derivatives. d-saccharic acid-1,4-lactone is one of the successful inhibitor^21^ of *β*-glucuronidase with IC_50_ = 3.6 µM^22^. Similarly, 2,5-di-*O*-acetyl-d-glucaro-1,4:6,3-Dilactone and d-glucurono-*γ*-lactone have also been studied with marked inhibition of the* β*-glucuronidase enzyme^23^. A piperazine diamine derivative has been found with IC_50_ = 0.08 µM^24,25^. It was suggested after SAR studies that the nucleophilic NH moiety impart greater efficacy to the molecule. Therefore, in recent study we are reporting the hydrazide derivative 2-amino-*N*′-[(*E*)-(4-hydroxy-3-methoxyphenyl)methylidene]benzohydrazide synthesized by reacting p-vanilline and 2-aminobenzohydrazide. Furthermore, substantial effects were observed by combining the active compounds with metal centers to produce metal complexes^26–34^. Metal complexes offer better activities over the conventional drugs^21,35–57^ as illustrated by copper (II) derivative of H–NMDP which revealed antiproliferation effect against A172 and LN229^58^. Similarly, aminoacid derived ligand H-HMAC and its Co(II), Ni(II), Cu(II) and Zn(II) metal complexes were studied for different enzyme inhibitory activities viz; urease, *α***-***Chymotrypsin,* Acetylcholinesterase (AChE) and butyrylcholinesterase^59^, and xanthine oxidase along with antioxidant potentials^60^. It was found that the ligand combined with zinc was found selectively inhibiting the urease enzyme with IC_50_ = 0.049 ± 0.01 µM. The same complex was also involved in inhibiting the xanthine oxidase with IC_50_ = 0.7 ± 0.01 µM. Therefore, it can be concluded that Schiff base metal complexes play a very important role in inhibiting enzymatic activities and biological activities. As a result, the current study is primarily concerned with synthesizing the metal complexes of the Schiff base ligand that is produced from hydrazides. The *β*-glucuronidase inhibition of the products was evaluated.

## Experimental

### Chemicals and reagents

Chemicals and solvents used in the current work were obtained in their purest forms from different local suppliers like Metal(II) acetates (where metal(II) = Co, Ni, Cu and Zn) from Riedel-de-Haen, Salicylaldehyde from Acros Organics, solvents from Merck and Sigma Aldrich. Metal salts were dried in a vacuum oven for 3–4 h at 80–100 °C before reaction. 2-aminobenzohydrazine was prepared using the previously reported procedure. The reaction environment was kept inert, and the manipulations were carried out in Schlenk line.

### Synthesis of N′-[(4-hydroxy-3-methoxyphenyl)methylidene] 2-aminobenzohydrazide (H-AHMB)

Benzohydrazide ligand was synthesized as described in the previously reported work^61^. 10 mmol of benzohydrazide was reacted 10 mmol of 4-hydroxy-3-methoxybenzaldehyde in 10 cm^3^ of distilled methanol and the mixture was stirred for about an hour. Yellow color solution was obtained which was concentrated using rotary evaporator. The single crystals were obtained from the concentrated solution in THF.

Yoeld: 84%, Color: Yellow, IR: cm^−1^ 3369 (s), 3215.34(b), 1641.42(s), 1616.35(m), 1604.77(s), 1489.05(m), 1456.26(s), 1408.04(s), 1361.74(s), 1300.02(s), 1234(s), 1203.58(w), 1170(s), 1153.43(s), 1097.50(s), 948.98(s), 925.83(m), 862.18(s), 855(s), 820(s), 770(s), 760(s), 748.38(s), 701(s), 696.30(s), 650.01(s), 590.22(s), 553.57(m). Elemental analysis: (C_22_H_19_N_3_O_2_) Calcd. C(63.15%), H(5.30%), N(14.73%), Found. C(63.69%), H(5.56%), N(14.46%), EI-MS: m/z (%) 286.1192 [C_15_H_16_N_3_O_3_]^+^, ^1^H-NMR 2.1(s, CH=N), 2.5(s,CDCl_3_), 3.4(s, NH_2_), 3.8(s, OCH_3_), 6.4(s, RCONH), 6.6(t, H17), 6.7(d, H4), 6.8(d, H16), 7.1(d, H19), 7.2(t, H18), 7.3(s, H2), 7.6(d, H5), 8.3 (s, OH), ppm. ^13^C{^1^H}-NMR 56.34 (OCH_3_),109.75 (CH, C17), 111.83 (C, C3), 115.44(CH, C18), 117.11(CH, C4), 122.81(CH, C2), 126.75(CH, C16), 132.90(CH, C19), 134.53 (C, C15), 148.23(C, C20), 148. 85(CH, C5), 149.62(CH, CH=N), 150.70(C, C–OH),165.93 (C, C=O)ppm.

### Synthesis of transition metal complexes {M-AHMB where M = Co^2+^, Ni^2+^, Cu^2+^, and Zn^2+^ as chlorides}

In a minimal volume of dry methanol, 30 mmol of the ligand (H-AHMB) was combined with 15 mmol of the salts of the divalent metals {Co(II), Ni(II), Cu(II), and Zn(II)}, and the combination was agitated for 6 to 8 h at room temperature. Following the development of metal complexes or adducts, they were filtered out and washed with dry n-hexane.

#### Cobalt adduct with N′-[(4-hydroxy-3-methoxyphenyl)methylidene] 2-aminobenzohydrazide [Co-AHMB]

Greyish green, Yield: 66%, IR: cm^−1^ 3369.64(s), 3180.62(b), 1641.42(s), 1616.35(m), 1604.77(s), 1489.05(m), 1456.26(s), 1408.04(s), 1361.74(s), 1300.02(s), 1240,23(s), 1200(w), 1161.15(w), 1153.43(s), 1097.50(s), 980(s), 950.91(s), 910(m), 867.97(s), 855(s), 820(s), 770(s), 760(s), 748.38(s), 701(s), 696.30(s), 650.01(s), 630.72(w), 590.22(s), 553.57(m), 499.56(s), 491.85(s), 459.06(s), 449.41(s), λ_max_ = 620 nm (ε = 16.0 M^−1^ cm^−1^, ^2^A_2_ → ^2^B_1_), *µ*_eff_ = 3.9 B.M.

#### Nickel adduct with N′-[(4-hydroxy-3-methoxyphenyl)methylidene] 2-aminobenzohydrazide [Ni-AHMB]

Emerald green, Yield: 74%, IR: cm^−1^ 3292.49(b), 1635(s), 1614.425(m), 1598.99(m), 1573.91(w), 1489.05(m), 1456.26(s), 1390.68(w), 1360(w), 1300(w), 1271.09(m), 1234.44(m), 1203.58(w), 1153.43(s), 1097.50(s), 980(s), 950.91(s), 910(m), 860.25(s), 855(m), 820(w), 760(w), 746.45(s), 721.38(w), 690.52(s), 646.15(w), 613.36(s), 576.72(s), 549.71(s), 499.56(s), 491(s), 462.92(s), λ_max_ = 710 nm (ε = 26.9 M^−1^ cm^−1^, ^1^A_1_ → ^1^A_2_), *µ*_eff_ = 2.9 B.M.

#### Copper adduct with N′-[(4-hydroxy-3-methoxyphenyl)methylidene] 2-aminobenzohydrazide [Cu-AHMB]

Dark green, Yield: 76%, IR: cm^−1^ 3600(b), 3290(m), 3000(m), 1610.56(s), 1600.92(s), 1571.99(w), 1537.27(s), 1489.05(m), 1463.97(s), 1442.75(s), 1430(s), 1379.10(s), 1360(w), 1300(w), 1290.38(s), 1246.02(s), 1193.94(s), 1151.50(s), 1097(s), 1053.13(s), 980(s), 950.91(s), 918(s), 873.75(s), 854.47(s), 817.82(s), 760(s), 746.45(s), 690.52(s), 646(s), 610(s), 596(s), 550(s), 490(s), λ_max_ = 820 nm (ε = 18.9 M^−1^ cm^−1^, ^2^E → ^2^T_2_), *µ*_eff_ = 1.9 B.M.

#### Zinc adduct with N′-[(4-hydroxy-3-methoxyphenyl)methylidene] 2-aminobenzohydrazide [Zn-AHMB]

Yellowish orange, Yield: 73%, IR: cm^−1^ 3369.64(s), 3219.19(b), 1639.49(s), 1616.35(s), 1604.77(s), 1489.05(s), 1456.26(s), 1435.04(s), 1408.04(s), 1359.82(s), 1300(w), 1271.09(m), 1238(s), 1230.58(w), 1203.58(s), 1170(s), 1153.43(s), 1097.50(s), 1037.70(s), 970(s), 948.98(s), 925.83(s), 867.97(s), 855(s), 820(s), 800(s), 750(s), 721.38(s), 694.37(s), 646.15(s), 630.72(m), 586.36(s), 553.57(s), 437.84(s).

### Instrumentation

Varian Elementar II instrument was used for finding the elemental composition (experimental). Vario 6, Analytic Jena atomic absorption spectrophotometer was used for the metal ion content. PerkinElmer spectrophotometer version 10.4.00 with serial number 95120 made in Lliantrisant, UK was used for recording the ATR spectra of all the samples. ^1^H-NMR and ^13^C-NMR spectra of the Hydrazide Schiff base ligand was found by BRUKER advance III 400 spectrometer.

### Crystal structure determination

A glass fiber was mounted with crystals of the ligand in innocuous paraffin oil. On oxford diffractometer using graphite-monochromated Mo-K radiation (λ = 0.71073), data were collected at 170 K. The structures were solved using Charge Flipping with the olex2.solve^62,63^ and refined with the olex2.refine^62^. The additional information file contains crystallographic information, and the structure was sent to the CCDC. via quoting the CCDC-number 2281686 for H-AHMB, data may be requested from the Cambridge Crystallographic Data Centre via FAX (+ 44-1223-336-033), email (deposit@ccdc.cam.ac.uk), or their online interface (at http://www.ccdc.cam.ac.uk).

### Hirshfeld surface analysis and 2D fingerprint plot

Crystal Explorer 21.5 was used for computing short contacts between neighbouring molecules using the refined cif of H-AHMB. Hirshfeld surfaces in the crystal lattice were investigated. The two-dimensional (2D) fingerprint plot of short interatomic contacts and the interaction energies were envisaged. The colour gradient (red to blue) were used to represent the parameters derived from the normalized contact distance *d*_norm_ described in terms of the van der Waals radii (vdW) of atoms, *d*_i_ and *d*_e_ parameters. The 2D finger plots derived between *d*_i_ and *d*_e_ within the range 0.6–2.8 Å were used to derive the set of interactions. Deep red colour on the represent the short interatomic interactions while the light red spots represent the poor interactions and the blue colour spots represent non-interacting regions. The Hirshfeld surface's distances from the neighboring atomic centers are displayed by the parameters used on 2D fingerprint plots between *di* and *de*.

### Biological studies

#### *β*-Glucuronidase inhibitory activity

*β*-Glucuronidase inhibitory activity by hydrazide Schiff base ligand (H-AHMB) and its metal (II) complexes was investigated using standard procedure. P-Nitrophenol produced as a result of reaction from the substrate during the procedure was measured spectrophotometrically at 405 nm to evaluate the *β*-glucuronidase activity. According to the procedure 185 µL of 0.1 M acetate buffer, 5 µL compound to be tested and 10 µL of enzyme solution were mixed and incubated at 37 °C for 30 min. SpectraMax plus 384, Molecular Devices, USA was used to read on a multiplate reader at 405 nm after the addition of 0.4 mM p-nitrophenyl-*β*-d-glucuronide (50 µL). All experiments were performed in triplicate.

#### 2,2-diphenyl-1-picrylhydrazyl) (DPPH) radical scavenging studies

The synthetic Schiff base ligand and its metal complexes were tested for their ability to scavenge DPPH radicals using the methodology described by Soare, Dinis, Cunha, and Almeida in 1997^64^. Initially, 1 mL of 0.2 mM DPPH dissolved in methanol was combined with 0.1 mL of each sample at 250, 500, 750, and 1000 g/mL concentrations. For 20 min, the reaction mixture was kept at 28 °C and kept in the dark. Methanol served as the control, which had all the components but no sample. A UV/Vis spectrophotometer was used to detect the absorbance at 517 nm in order to assess the DPPH radical scavenging activity. For comparison, the DPPH radical scavenging capacity of ascorbic acid was also determined.

$$\% \;Inhibition\;effect = \frac{{A_{c} - A_{s} }}{{A_{c} }} \times 100$$

A_c_ = Absorbance by the control, A_s_ = Absorbance by the sample^37^.

## Results and discussion

The ligand H-AHMB and its metal derivatives were synthesized and characterized using different analytical and spectroscopic techniques. The elemental composition is in agreement with the formation of the ligand H-AHMB after the reaction of vanillin with benzohydrazide. The percent composition of the complexes revealed the formation of the metal derivatives of the H-AHMB ligand. Mass spectrum of the H-AHMB (as shown in supplementary file) also revealed the molecular ion peak at the m/z = 286.1201 for [C_15_H_16_N_3_O_3_]^+^.

### Structural studies of H-AHMB ligand

**Figure 1:**
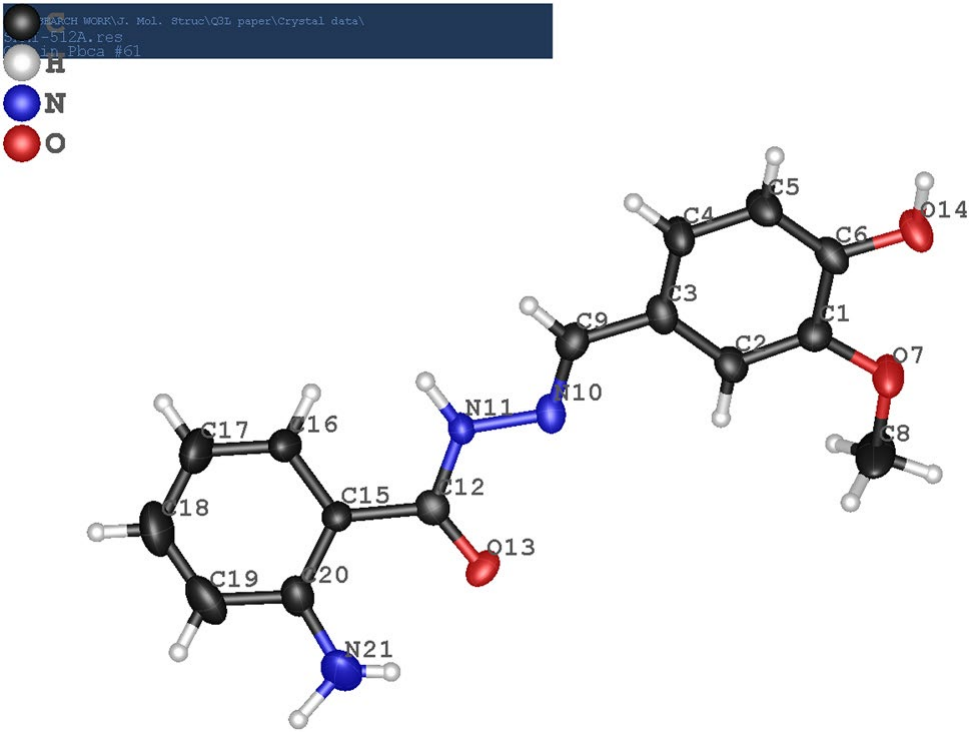
Molecular structure of H-AHMB.

The single crystals of the synthesized hydrazide derived Schiff base ligand H-AHMB was structurally characterized using single crystal diffraction analysis (Fig. 1). The crystal data as given in Table 1 revealed orthorhombic, *Pbca* space group. The Schiff base linkage in H-AHMB {C4-N10 = 1.2946(6) Å (Table 2)} is comparatively longer than similar bond lengths in amino acid derivative (H-HMAC) with C9-N8 = 1.2910 Å^60^ and in cyclized hydrazide derivative (H-HHAQ) with C8-N9 = 1.275(9)Å^61^.

**Table 1:** Crystal data and structure refinement parameters for H-AHMB.

Compound	H-AHMB
Molecular formula	C_15_H_15_N_3_O_3_
Formula mass	898.91
Temp.	170(2) K
λ	0.7107 Å
Crystal system, space group	Orthorhombic, *Pbca*
Unit cell dimensions	a = 16.092 (2) Å, α = 90° b = 7.9264 (8) Å, β = 90° c = 22.901 (3) Å, γ = 90°
V	2921.0 (6) Å^3^
Θ range for data collection	3.1–27.0°
Limiting indices	0 < = h < = 19, 0 < = k < = 10, 0 < = l < = 20
Measured reflections	5182
Independent reflections	2750
R_int_	0.076
Refinement method	Full-matrix least-squares on F^2^
Data/restraints/parameters	2750/0/217
*R*[*F*^2^ > 2σ(*F*^2^)]	0.092, wR(F^2^) = 0.177

**Table 2:** Selected bond lengths and bond angles of the H-AHMB (Å, °).

Selected bond lengths of the H-AHMB Schiff base ligand			
Moiety	Bond length, Å	Moiety	Bond length, Å
O13—C12	1.248 (5)	O7—C1	1.375 (5)
O14—C6	1.360 (5)	O7—C8	1.425 (7)
N10—C9	1.294 (6)	C12—C15	1.474 (6)
C20—C19	1.410 (7)	C15—C16	1.397 (6)
C20—N21	1.362 (7)	C3—C2	1.400 (6)
N11—N10	1.389 (5)	C3—C9	1.450 (7)
N11—C12	1.341 (6)	C3—C4	1.388 (6)

Selected bond angles of the H-AHMB Schiff base ligand			
Moiety	Bond angle, °	Moiety	Bond angle, °
C12—N11—N10	119.1 (4)	N10—C9—C3	122.4 (4)
C4—C3—C2	119.3 (5)	C5—C6—O14	123.6 (5)
C4—C3—C9	118.4 (4)	C1—C6—O14	117.0 (4)
C9—N10—N11	114.2 (4)	C8—O7—C1	117.2 (4)
C19—C20—C15	118.1 (5)	O7—C1—C2	125.1 (4)
N21—C20—C15	123.8 (5)	O7—C1—C6	113.9 (4)
N21—C20—C19	118.0 (6)	C15—C12—N11	115.3 (4)
N11—C12—O13	122.0 (4)	C15—C12—O13	122.7 (4)

**Figure 2:**
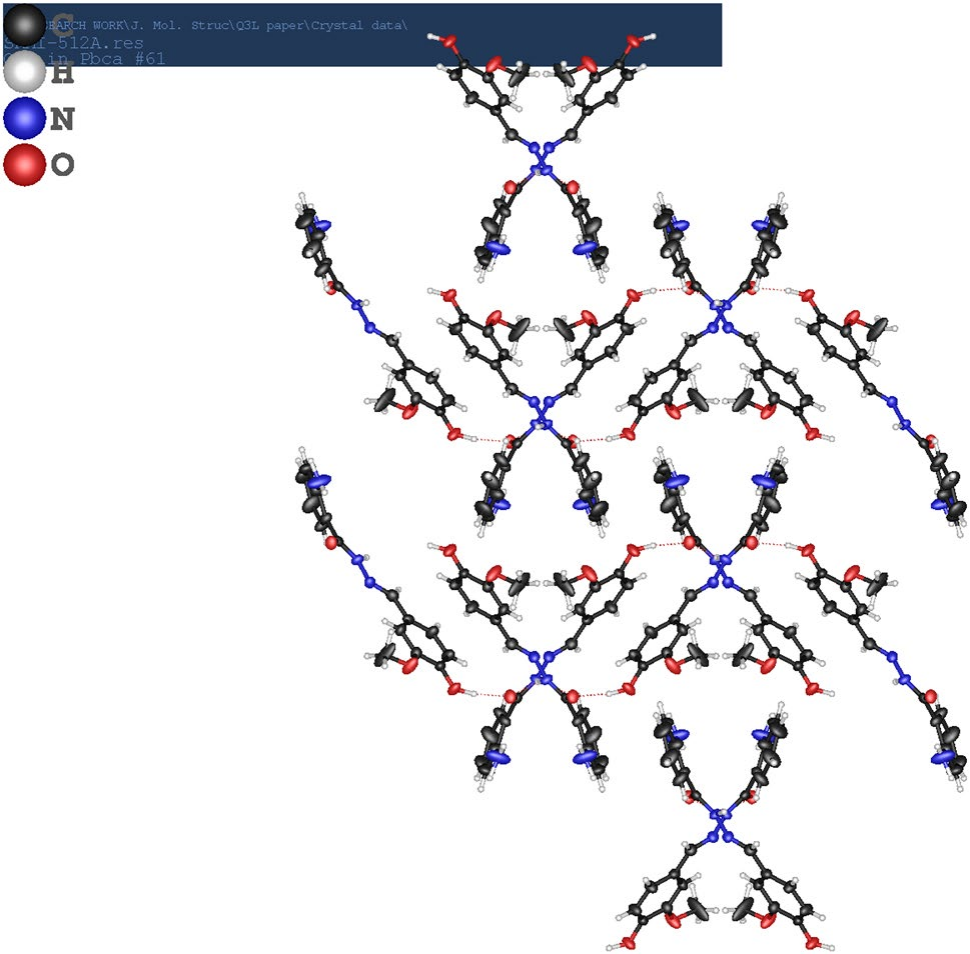
Crystal packing diagram of H-AHMB along b.

The plane produced by C12-C15-C20-C19-C18-C17-C16 is twisted by 44.35° than the plane produced by C1-C2-C3-C4-C5-C6-C9-N10-N11 due to the formation of Schiff base linkage at C9-N10 moiety. This twist is also responsible for the comparative longer bond distance of the C=N moiety. The bond angle for C12—N11—N10 is 119.1 (4)° and for N10—C9—C3 is 122.4 (4)° is also supporting the aforementioned twist. Further support is also revealed from the bond angle of C4—C3—C9 which is 118.4 (4)° as may be seen in Table 2. The crystal lattice is produced by intermolecular hydrogen bonding of 1.745 Å between O14-H14…..O13 comparatively longer than similar bond distance as may be seen in amino acid derivative (H-HMAC) with a distance of 1.687 Å for O22-H23. The crystal packing diagram is shown in Fig. 2.

### NMR study

The ligand H-AHMB was also characterized using ^1^H-NMR and ^13^C-NMR (figure 1S and 2S). ^1^H-NMR of H-AHMB revealed representative peaks at their respective positions of the spectrum. A singlet at 2.1 ppm was assigned to the azomethine linkage. Another singlet at 3.4 ppm was assigned to the amine group. The amide (NH) is also observed as a singlet at 6.4 ppm. The aromatic protons were clearly identified by observation at their respective places. The ^13^C-NMR was also assigned without any constrains to the respective positions. The six quaternary carbon nuclei were observed viz; C=O was observed at 165.9 ppm, C–OH was seen at 150 ppm position and the Schiff base linkage was seen at 149.6 ppm. Rest of the spectrum is assigned to the respective carbon atoms.

### Vibrational study

**Scheme 2:**
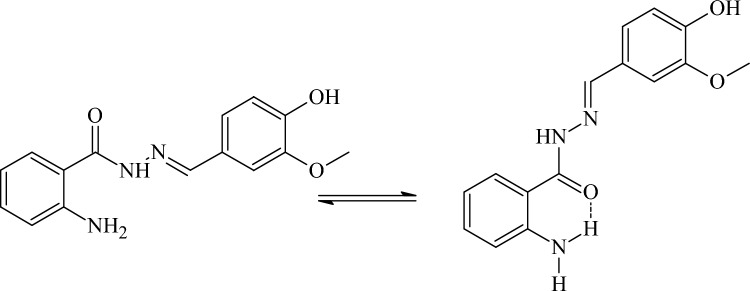
Intermolecular hydrogen bonding in H-AHMB.

IR spectroscopic analysis was also performed to reveal the vibrations (figure 4S-8S). A sharp band at 3369 cm^−1^ was observed suggesting the involvement of –NH_2_ moiety in the intramolecular hydrogen bonding as shown in Scheme 2.

Imine-iminium type of tautomerism is seen which lead to the vibration of NH only^46^. Hydroxyl group of vanillin is observed as a broad band at 3200 cm^−1^. A vibrational mode for C=N is observed at 1641 cm^−1^ as a strong band. Whereas the NH_2_ bends were observed as two bands at positions like 1616 cm^−1^ and 1604 cm^−1^. The CH_3_ bend is observed at 1440 cm^−1^ as medium band. The OH bend in plane was seen at 1350 cm^−1^ as a medium band and the out of the plane bend was observed at 730 cm^−1^ as a weak band.

**Figure 3:**
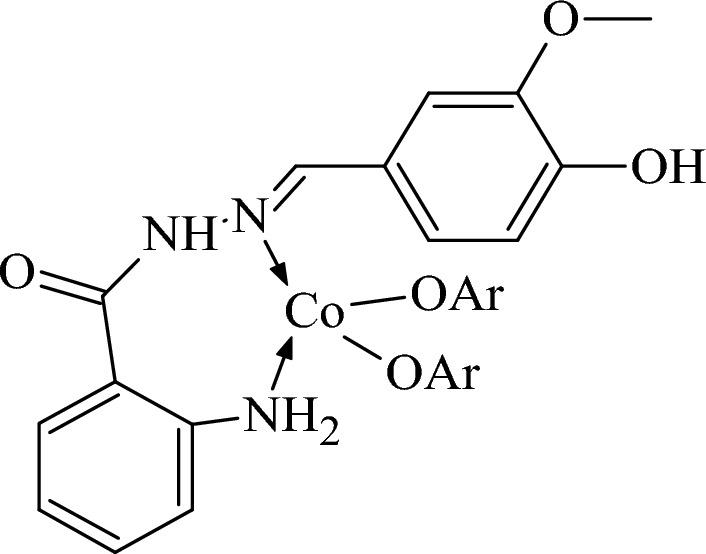
Co-AHMB complex.

All other bands were seen at their respective positions except for the moieties involved in coordination with metal centers. In case of Co-AHMB complex the NH_2_ bands were observed at 3369 cm^−1^ and with shoulder peak whereas the band at 3216 cm^−1^ was displaced to 3180 cm^−1^ in Co-AHMB. Similarly, the ketone vibrational band was found missing in the Co-AHMB metal complex. The C=N band has been observed at 1641 cm^−1^ as was in ligand. Most likely the coordination in case of Co-AHMB complex may be assigned to the C=N and –OH groups as shown in Fig. 3.

Hence a kind of coordination polymer may be seen with distorted square planar /tetrahedral orientation. The structure may be concluded exactly upon successful crystallization from saturated solution. Till now all the attempts toward crystal growth were not fruitful.

Representative bands in case of Ni-AHMB complex were displaced to lower wavenumbers representing coordination. These include bands at 1641 cm^−1^ which have been affected to a greater extent and is converted to weak band. Similarly, the two pairs of bands have been displaced to 1614 cm^−1^ and 1598 cm^−1^ revealing ∆λ = 2 to 6 cm^−1^. The –OH band has also been effected and look absent or submerged in the region of 3200–3400 cm^−1^. These two observations are pointing toward the coordination through NH_2_ and C=N group. This conclusion is further supported by disappearance of shoulder peak at 3200 cm^−1^. Overall, coordination in case of Ni–AHMB complex is like the Co-AHMB complex.

Cu-AHMB complex with the hydrazide Schiff base ligand is not different than the cobalt and nickel derivatives. The differences seen here in Cu-AHMB complex were absence or broadening of –OH band, the absence of band responsible for C=N and the shift in the pair of bands {1610 cm^−1^ and 1600 cm^−1^}. The bands responsible for in plane and out plane bends of –OH are completely absent. Zn-complex is exactly similar in coordination with Schiff base ligand as was Co-AHMB complex. Therefore, no need for further explanation. All the metal complexes were studied for elemental composition and close alignment of the calculated and found values show the formation of synthesized complexes.

### Hirshfeld surface analysis

The intermolecular interactions in the crystal of H-AHMB can be affirmed from studying the Hirshfeld surface analysis. Intermolecular interactions are of immense importance in studying enzymatic interactions of the test compound and the active sites. Stronger intermolecular interactions in the crystal lattice is shown as red patches on the *d*_*norm*_ surface plot. The computation of the *d*_*norm*_ surface plot is done by measuring the *d*_*e*_ (external)and *d*_*i*_ (internal) distances to the nearby atoms.

**Figure 4:**
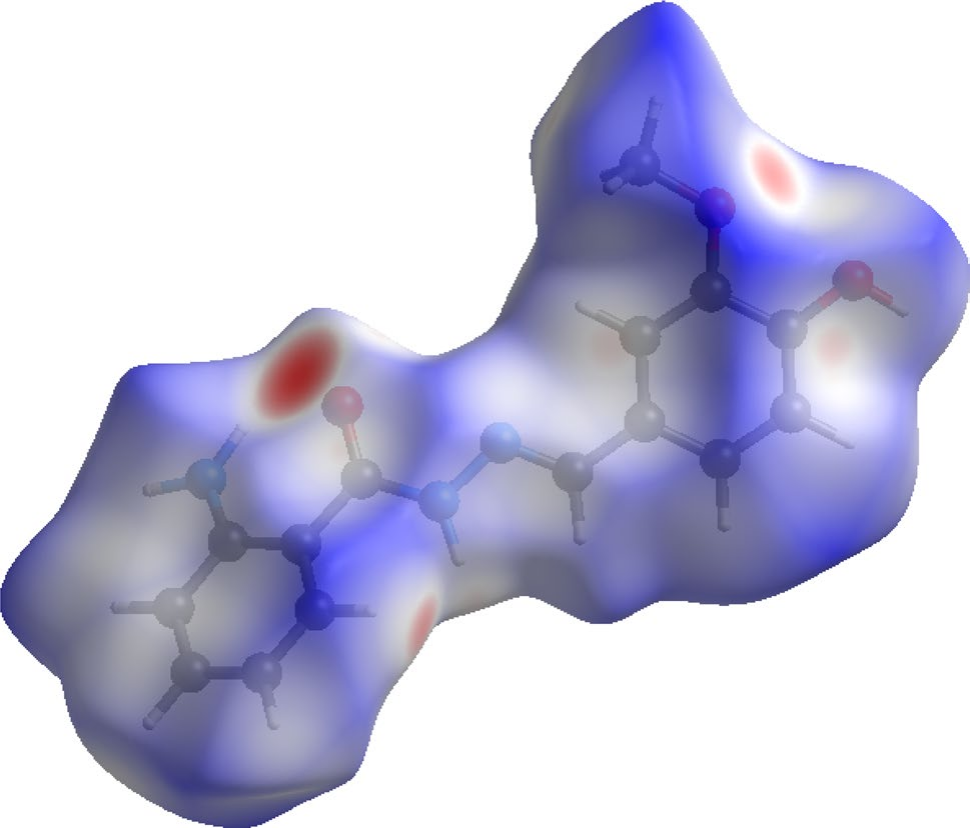
*d*_norm_ plot decorated Hirshfeld surface in different direction of lattice of H-AHMB.

**Figure 5:**
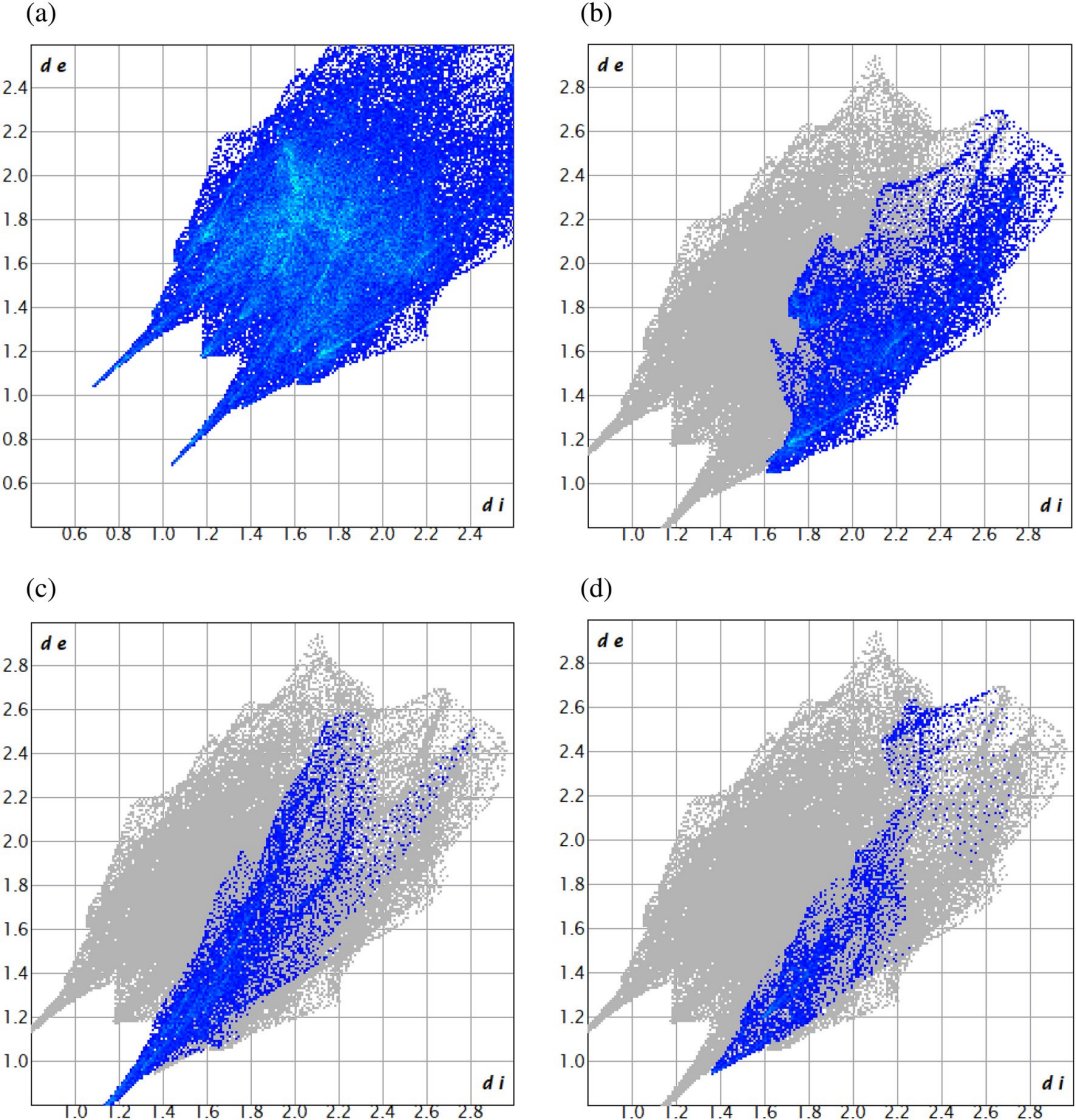
2D fingerprint plots of short intermolecular interaction with their associate contributions to the HS in the crystal lattice of H-AHMB (**a**) all atoms interactions (**b**) C ···H/H ···C (**c**) O ···H/H ···O (d) N ···H/H ···N.

The Hirshfeld surface area of H-AHMB ligand covers area of 346.70 Å^2^ and spread over 357.41 Å^3^ volume. The isovalue was found to be 0.5 with 0.701 globularity. The scaled colored patched index of H-AHMB can be seen in Fig. 4 whereas the quantitative and envisage of 2D fingerprint plot may be seen in Fig. 5. The short atomic contacts H···H = 39.9%, C···C = 2.6%, C···H = 15.8%, C···N = 0.5%, and H···O = 9.6% suggest that the major supports are from H···H, C···H and H···O compared to the other atomic interactions^65–70^.

### β-Glucuronidase inhibition

New metal based inhibitor particularly designed by combining essential metals with the organic substrates will definitely help to overcome the effects as mentioned in “Introduction” section caused by overly expressed *β*-glucuronides enzyme. Obviously such an inhibitor will reveal the value of metal ions as a novel strategy to overcome the overly expressed *β*-glucuronides enzyme. Herein, we are reporting the inhibition of *β*-glucuronidase enzyme by the metal complex derivatives of N′-[(4-hydroxy-3-methoxyphenyl)methylidene]-2-aminobenzohydrazide (H-AHMB). The results are tabulated in Table 3 and compared with d-saccharic acid-1,4-lactone. The Schiff base ligand H-AHMB and its metal derivatives were found active against the *β*-glucuronidase enzyme. Comparing the activity of the ligand with the metal derivatives it may be clarified that metal derivatives are more active than the neat ligand. From cobalt to zinc all the metal derivatives reveal similar activities. Interestingly, the inhibitory activity of all the metal derivatives are more than the standard drug used. It is apparent that upon complexation the activity is more enhanced. The nature of metal ion is also affecting the activity. For example zinc is enhancing the inhibition more than the other metal ions. Similarly, cobalt is following the zinc in inhibitory activity. By comparison, it is clear that Zn-AHMB is almost three times more effective than standard drug already in use. Whereas, Co-AHMB is active more than two folds against *β*-glucuronides enzyme in comparison to the standard drug. Other, metal complexes like Cu-AHMB is also very active with IC_50_ = 32.3 ± 1.00 µM. And Ni-AHMB is the least active metal complex. Activity of Zn-AHMB may be linked to the structural features of *β*-glucuronidase enzyme. The *β*-glucuronidase enzyme bears two glutamic acid as active sites, zinc center is more Lewis acidic than the other metal ions and can actively react with O-containing sites hence, stopping the activity^71^. Similarly, cobalt is present in + 2 oxidation state which bears less Lewis acidity than zinc center, therefore activity is less pronounced^71^. The less activity of nickel may be linked to its less binding potentials to carboxylic acid moieties in comparison to N-terminus amino acids and other nitrogenous substrates.

**Table 3:** *β-*Glucuronidase inhibition by H-AHMB and its metal derivatives.

Compound	Conc. (mM)	% inhibition	IC_50_ ± SEM (µM)
H-AHMB	0.05	86.2	280.6 ± 2.02
Co-AHMB	0.05	78.8	21.7 ± 2.54
Ni-AHMB	0.05	96.0	40.59 ± 1.20
Cu-AHMB	0.05	73.3	32.3 ± 1.00
Zn-AHMB	0.05	98.3	17.3 ± 0.68
d-saccharic acid-1,4-lactone	0.05	89.4	45.75 ± 2.16

In conclusion, metal based *β*-glucuronidase inhibitor can be designed through the reaction of hydrazide based Schiff base ligand with essential metal ions.

### Free radical scavenging activities

Free radicals are short lived species bearing unpaired electrons and are usually produced inside mitochondria. Major free radicals are peroxide, superoxide ions, nitrates or peroxy nitrates. Many biological problems like ageing, rheumatoid arthritis, AIDS, cancer and neurodegenerative diseases are linked to free radicals or their effects^72^. Therefore, stopping their activities will be helpful in overcoming many diseases. Developing a successful antioxidant is an active area of research. Antioxidant substances bear hydrogen atoms or peroxide decomposers or oxygen quencher. Biological systems are supplied with natural antioxidants like glutathione, vitamin C, superoxide dismutases etc. Metal derivatives are supposed to be the better antioxidants which will definitely play role in increasing shelf life of industrial products.

Therefore, here we are also finding out the antioxidant potential of H-AHMB and its metal complexes against DPPH free radicals. Table 4 reveal the antioxidant potential of H-AHMB and its metal complexes. The ligand H-AHMB is inactive on its own whereas, except Zn-AHMB all the metal derivatives shown DPPH free radical scavenging activities. Among the active metal derivatives Co-AHMB with IC_50_ = 98.2 ± 1.78 µM is the most active complex whereas, other metal derivatives were found weakly active. The activity of cobalt containing complex may be linked to its structural features where the C=N moiety seems to be free and may be involved in abstracting the proton of DPPH free radical^73^. The activity of Co-AHMB complex is less than the activity shown by gallic acid used as standard.

**Table 4:** DPPH scavenging activity by H-AHMB and its metal derivatives.

Compound	Conc. (mM)	% Radical scavenging activity	IC_50_ ± SEM (µM)
H-AHMB	0.05	22.97	–
Co-AHMB	0.05	88.46	98.2 ± 1.78
Ni-AHMB	0.05	52.40	467 ± 2.90
Cu-AHMB	0.05	84.29	183 ± 6.60
Zn-AHMB	0.05	49.16	–
Gallic acid	0.05	93.13	63.0 ± 0.43

## Conclusion

The ligand N′-[(4-hydroxy-3-methoxyphenyl)methylidene]-2-aminobenzohydrazide (H-AHMB) was synthesized from O-vanillin and 2-aminobenzohydrazide. It had been fully and well characterized prior to its complexation with metal ions like Co^2+^, Ni^2+^, Cu^2+^ and Zn^2+^. The characterization techniques included FTIR, high resolution ESI(+) mass spectral analysis, ^1^H and ^13^C-NMR and single crystal diffraction analysis. All the spectral analysis confirm the formation of the ligand H-AHMB. The crystal data revealed orthorhombic, *Pbca* space group. Hirshfeld surface analysis revealed major interactions H…H, O…H and C…H confirming the hydrogen bonds in H-AHMB. The metal derivatives were prepared by reacting metal (II) chlorides with H-AHMB ligand. Elemental analyses, FTIR and UV/vis studies were carried out to confirm the metal and H-AHMB bonding. Single and diffractable crystals cannot be grown therefore, complete structural analysis will be reported in case of crystal growth.* β*-glucuronidase enzyme inhibition and DPPH free radical scavenging capabilities were also studied. Among the synthesized compounds Zn-AHMB with IC_50_ = 17.3 ± 0.68 µM was found with greater efficiency than the standard drug in inhibiting the *β*-glucuronidase enzyme. Whereas, in DPPH radical scavenging capabilities Co-AHMB with IC_50_ = 98.2 ± 1.78 µM was found most active in stopping DPPH free radicals. But the activity of Co-AHMB was found less than the standard drug. Overall, metallo *β*-glucuronidase inhibiting drug can be designed successfully using the H-AHMB ligand.

### Supplementary Information

Supplementary Information.

Supplementary Figures.

## Data Availability

NMR and IR spectral data is contained within the supplementary material. Crystallographic details are supplied in the supplementary information file and the structure was submitted to the CCDC. Data can be obtained free of charge from the Cambridge Crystallographic Data Centre by FAX (+ 44-1223-336-033), email (deposit@ccdc.cam.ac.uk) or their web interface (at http://www.ccdc.cam.ac.uk) by quoting the CCDC-number 2281686 for H-AHMB.
