# Supplementary material for: Synthesis, characterization, Hirshfeld surface analysis, antioxidant and selective β-glucuronidase inhibitory studies of transition metal complexes of hydrazide based Schiff base ligand
Source: Sci Rep. 2024 Jan 4;14:515. doi: 10.1038/s41598-023-49893-6 (PMC10766943; doi:10.1038/s41598-023-49893-6)

checkCIF/PLATON report

Structure factors have been supplied for datablock(s) SRMI-512A

THIS REPORT IS FOR GUIDANCE ONLY. IF USED AS PART OF A REVIEW PROCEDURE FOR PUBLICATION, IT SHOULD NOT REPLACE THE EXPERTISE OF AN EXPERIENCED CRYSTALLOGRAPHIC REFEREE.

No syntax errors found. CIF dictionary Interpreting this report

Datablock: SRMI-512A

Bond precision: C-C = 0.0068 A Wavelength=0.71073

Cell:	a=16.092(2)	b=7.9264(9)	c=22.901(3)
	alpha=90	beta=90	gamma=90
Temperature:	0 K		

	Calculated	Reported
Volume	2921.1(6)	2921.0(6)
Space group	P b c a	P b c a
Hall group	-P 2ac 2ab	-P 2ac 2ab
Moiety formula	C15 H15 N3 O3	C15 H15 N3 O3
Sum formula	C15 H15 N3 O3	C15 H15 N3 O3
Mr	285.30	287.32
Dx,g cm-3	1.298	1.307
Z	8	8
Mu (mm-1)	0.093	0.093
F000	1200.0	1216.8
F000'	1200.55	
h,k,lmax	20,10,29	19,10,28
Nref	3201	2750
Tmin,Tmax		0.776,1.000
Tmin'		

Correction method= # Reported T Limits: Tmin=0.776 Tmax=1.000
AbsCorr = MULTI-SCAN

Data completeness= 0.859 Theta(max)= 27.060

R(reflections)= 0.0771(1215) wR2(reflections)= 0.1642(2750)

S = 0.980 Npar= 218

The following ALERTS were generated. Each ALERT has the format
test-name_ALERT_alert-type_alert-level.
Click on the hyperlinks for more details of the test.

Alert level A

```
EXPT005_ALERT_1_A  _exptl_crystal_description is missing
            Crystal habit description.
            The following tests will not be performed.
            CRYSR_01
PLAT197_ALERT_1_A Missing _cell_measurement_temperature Datum ....     Please Add
PLAT198_ALERT_1_A Missing _diffrn_ambient_temperature   Datum ....     Please Add
PLAT699_ALERT_1_A Missing _exptl_crystal_description Value .......     Please Do !
```

Alert level B

```
PLAT097_ALERT_2_B Large Reported Max.  (Positive) Residual Density       0.85 eA-3
PLAT196_ALERT_1_B No TEMP record and _measurement_temperature .NE.        293 Degree
PLAT245_ALERT_2_B U(iso) H21A      Smaller than U(eq) N21       by      0.060 Ang**2
```

Alert level C

```
CHEMW01_ALERT_1_C  The difference between the given and expected weight for
            compound is greater 1 mass unit. Check that all hydrogen
            atoms have been taken into account.
DIFMN02_ALERT_2_C  The minimum difference density is < -0.1*ZMAX*0.75
            _refine_diff_density_min given =     -0.781
            Test value =     -0.600
DIFMN03_ALERT_1_C  The minimum difference density is < -0.1*ZMAX*0.75
            The relevant atom site should be identified.
DIFMX02_ALERT_1_C  The maximum difference density is > 0.1*ZMAX*0.75
            The relevant atom site should be identified.
SHFSU01_ALERT_2_C  The absolute value of parameter shift to su ratio > 0.05
            Absolute value of the parameter shift to su ratio given   0.051
            Additional refinement cycles may be required.
PLAT026_ALERT_3_C Ratio Observed / Unique Reflections (too) Low ..        44% Check
PLAT029_ALERT_3_C _diffrn_measured_fraction_theta_full value Low .      0.968 Why?
PLAT043_ALERT_1_C Calculated and Reported Mol. Weight Differ by ..       2.02 Check
PLAT053_ALERT_1_C Minimum Crystal Dimension Missing (or Error) ...     Please Check
PLAT054_ALERT_1_C Medium  Crystal Dimension Missing (or Error) ...     Please Check
PLAT055_ALERT_1_C Maximum Crystal Dimension Missing (or Error) ...     Please Check
PLAT068_ALERT_1_C Reported F000 Differs from Calcd (or Missing)...     Please Check
PLAT098_ALERT_2_C Large Reported Min.  (Negative) Residual Density      -0.78 eA-3
PLAT213_ALERT_2_C Atom C8              has ADP max/min Ratio .....        3.5 prolat
PLAT220_ALERT_2_C NonSolvent   Resd 1  C   Ueq(max)/Ueq(min) Range        3.3 Ratio
PLAT222_ALERT_3_C NonSolvent Resd 1  H   Uiso(max)/Uiso(min) Range       10.0 Ratio
PLAT245_ALERT_2_C U(iso) H8A       Smaller than U(eq) C8        by      0.016 Ang**2
PLAT250_ALERT_2_C Large U3/U1 Ratio for Average U(i,j) Tensor ....        2.1 Note
PLAT340_ALERT_3_C Low Bond Precision on  C-C Bonds ...............    0.00679 Ang.
PLAT351_ALERT_3_C Long   C-H (X0.96,N1.08A)  C8       - H8C      .       1.12 Ang.
PLAT352_ALERT_3_C Short  N-H (X0.87,N1.01A)  N21      - H21A     .       0.76 Ang.
PLAT353_ALERT_3_C Long   N-H (N0.87,N1.01A)  N21      - H21B     .       1.01 Ang.
PLAT355_ALERT_3_C Long   O-H (X0.82,N0.98A)  O14      - H14      .       1.01 Ang.
PLAT906_ALERT_3_C Large K Value in the Analysis of Variance ......     22.853 Check
PLAT906_ALERT_3_C Large K Value in the Analysis of Variance ......      4.784 Check
```

```
PLAT906_ALERT_3_C Large K Value in the Analysis of Variance ......      2.818 Check
PLAT911_ALERT_3_C Missing FCF Refl Between Thmin & STh/L=    0.600         80 Report
```

Alert level G

```
PLAT769_ALERT_4_G CIF Embedded explicitly supplied scattering data     Please Note
PLAT910_ALERT_3_G Missing # of FCF Reflection(s) Below Theta(Min).          4 Note
PLAT912_ALERT_4_G Missing # of FCF Reflections Above STh/L=  0.600        353 Note
PLAT960_ALERT_3_G Number of Intensities with I  <   - 2*sig(I) ...          4 Check
PLAT978_ALERT_2_G Number C-C Bonds with Positive Residual Density.          1 Info
```

```
   4 ALERT level A = Most likely a serious problem - resolve or explain
   3 ALERT level B = A potentially serious problem, consider carefully
  27 ALERT level C = Check. Ensure it is not caused by an omission or oversight
   5 ALERT level G = General information/check it is not something unexpected

  13 ALERT type 1 CIF construction/syntax error, inconsistent or missing data
  10 ALERT type 2 Indicator that the structure model may be wrong or deficient
  14 ALERT type 3 Indicator that the structure quality may be low
   2 ALERT type 4 Improvement, methodology, query or suggestion
   0 ALERT type 5 Informative message, check
```

It is advisable to attempt to resolve as many as possible of the alerts in all categories. Often the minor alerts point to easily fixed oversights, errors and omissions in your CIF or refinement strategy, so attention to these fine details can be worthwhile. In order to resolve some of the more serious problems it may be necessary to carry out additional measurements or structure refinements. However, the purpose of your study may justify the reported deviations and the more serious of these should normally be commented upon in the discussion or experimental section of a paper or in the "special_details" fields of the CIF. checkCIF was carefully designed to identify outliers and unusual parameters, but every test has its limitations and alerts that are not important in a particular case may appear. Conversely, the absence of alerts does not guarantee there are no aspects of the results needing attention. It is up to the individual to critically assess their own results and, if necessary, seek expert advice.

Publication of your CIF in IUCr journals

A basic structural check has been run on your CIF. These basic checks will be run on all CIFs submitted for publication in IUCr journals (*Acta Crystallographica*, *Journal of Applied Crystallography*, *Journal of Synchrotron Radiation*); however, if you intend to submit to *Acta Crystallographica Section C* or *E* or *IUCrData*, you should make sure that full publication checks are run on the final version of your CIF prior to submission.

Publication of your CIF in other journals

Please refer to the *Notes for Authors* of the relevant journal for any special instructions relating to CIF submission.

Validation response form

Please find below a validation response form (VRF) that can be filled in and pasted into your CIF.

```
# start Validation Reply Form
_vrf_EXPT005_SRMI-512A
;
PROBLEM: _exptl_crystal_description is missing
RESPONSE: ...
;
_vrf_PLAT197_SRMI-512A
;
PROBLEM: Missing _cell_measurement_temperature Datum ....     Please Add
RESPONSE: ...
;
_vrf_PLAT198_SRMI-512A
;
PROBLEM: Missing _diffrn_ambient_temperature   Datum ....     Please Add
RESPONSE: ...
;
_vrf_PLAT699_SRMI-512A
;
PROBLEM: Missing _exptl_crystal_description Value .......     Please Do !
RESPONSE: ...
;
# end Validation Reply Form
```

PLATON version of 28/11/2022; check.def file version of 28/11/2022

Datablock SRMI-512A - ellipsoid plot

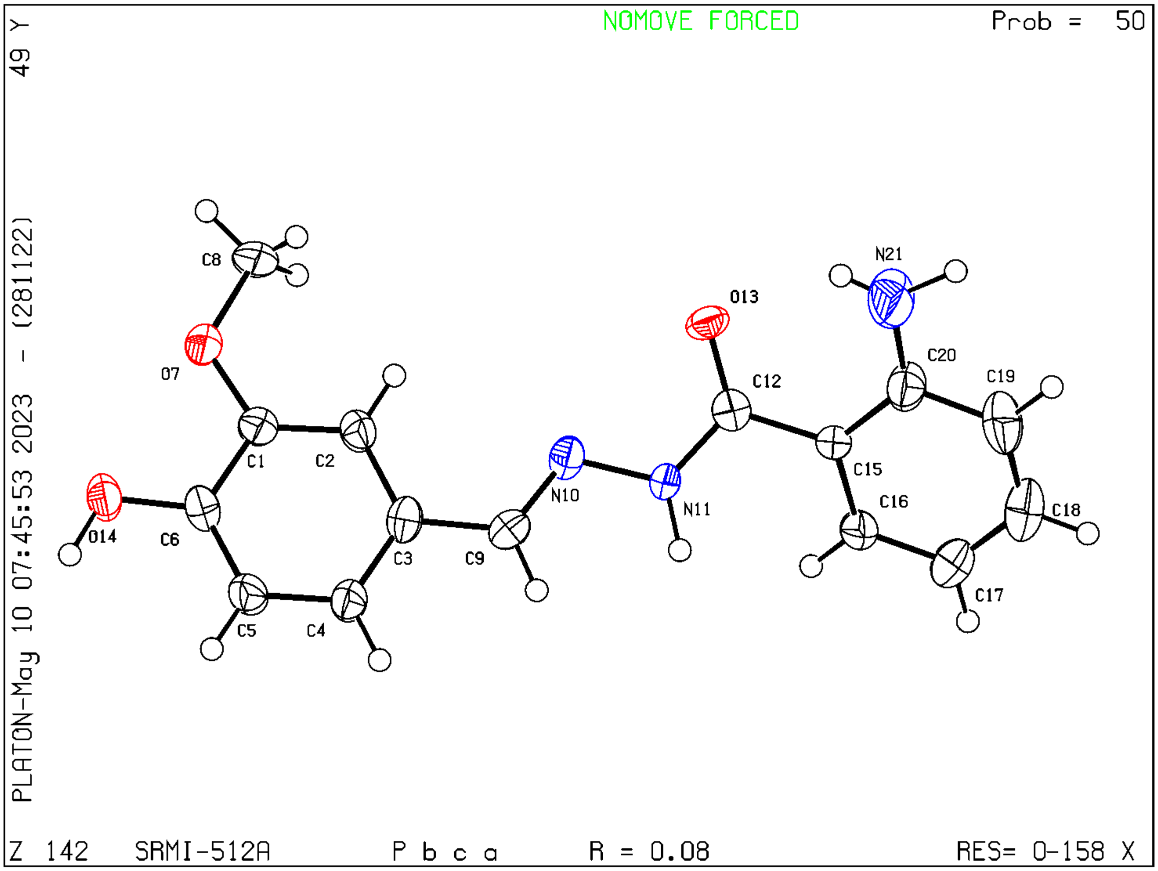

Supplement: Supplementary file 1 — Supplementary Information. [file 41598_2023_49893_MOESM1_ESM.pdf]
