# Supplementary material for: Synthesis, characterization, Hirshfeld surface analysis, antioxidant and selective β-glucuronidase inhibitory studies of transition metal complexes of hydrazide based Schiff base ligand
Source: Sci Rep. 2024 Jan 4;14:515. doi: 10.1038/s41598-023-49893-6 (PMC10766943; doi:10.1038/s41598-023-49893-6)
Supplement: Supplementary file 2 — Supplementary Figures. [file 41598_2023_49893_MOESM2_ESM.docx]

**Supplementary Information**

**Synthesis, characterization, Hirshfeld surface analysis, antioxidant and selective *β*-glucuronidase inhibitory studies of transition metal complexes of hydrazide based Schiff base ligand**

**Farzia^a^, Sadia Rehman*^a^, Muhammad Ikram**^a^, Adnan Khan***^b^, Rizwan Khan^c^, Mutasem Omar Sinnokrot^d^, Momin Khan^a^, Abdullah F. AlAsmari^e^, Fawaz Alasmari^e^, Metab Alharbi^e^**

^a^ Department of Chemistry, Abdul Wali Khan University, Mardan Pakistan

^b^ School of Physics & the Key Laboratory of Weak Light Nonlinear Photonics, Ministry of Education, Nankai University, Tianjin, 300071, People’s Republic of China

^c^ Department of Zoology, Abdul Wali Khan University, Mardan Pakistan

^d^ College of Arts and Sciences, American University of Iraq-Baghdad, Airport Road Baghdad, Iraq

^e^ Department of Pharmacology and Toxicology, College of Pharmacy, King Saud University, Riyadh 11451, Saudi Arabia.

*E-mail addresses:* sadia@awkum.edu.pk (S. Rehman), ikram@awkum.edu.pk (M. Ikram), [adnanphyzx@nankai.edu.cn](mailto:adnanphyzx@nankai.edu.cn) (A. Khan)

**
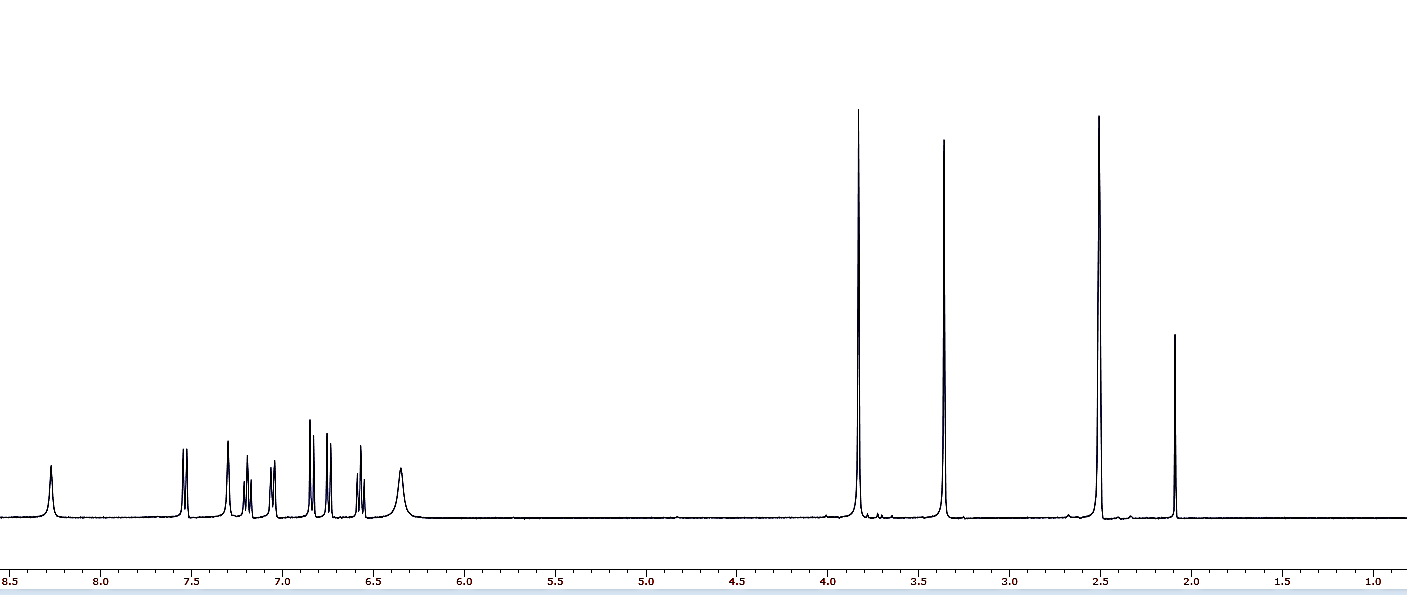
**

**Figure 1S: ^1^H-NMR of H-AHMB**

**
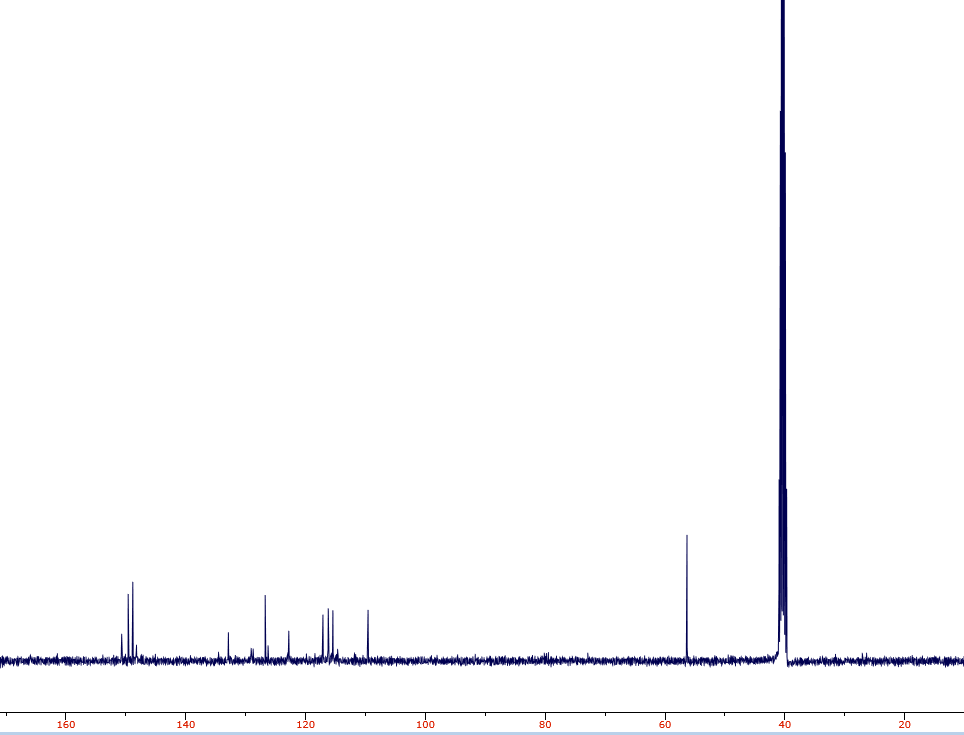
**

**Figure 2S: ^13^C-NMR of H-AHMB**

**
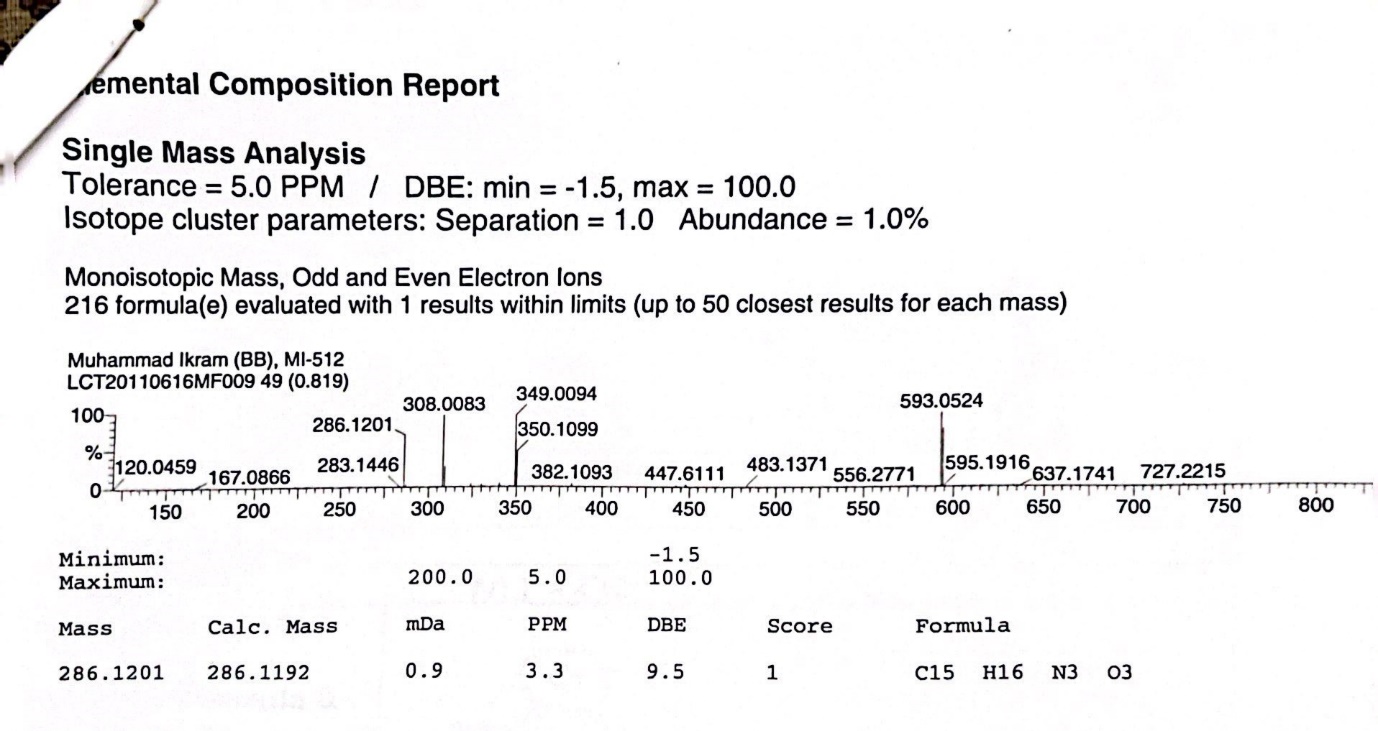
**

**Figure 3S: Mass spectrum of H-AHMB**

**
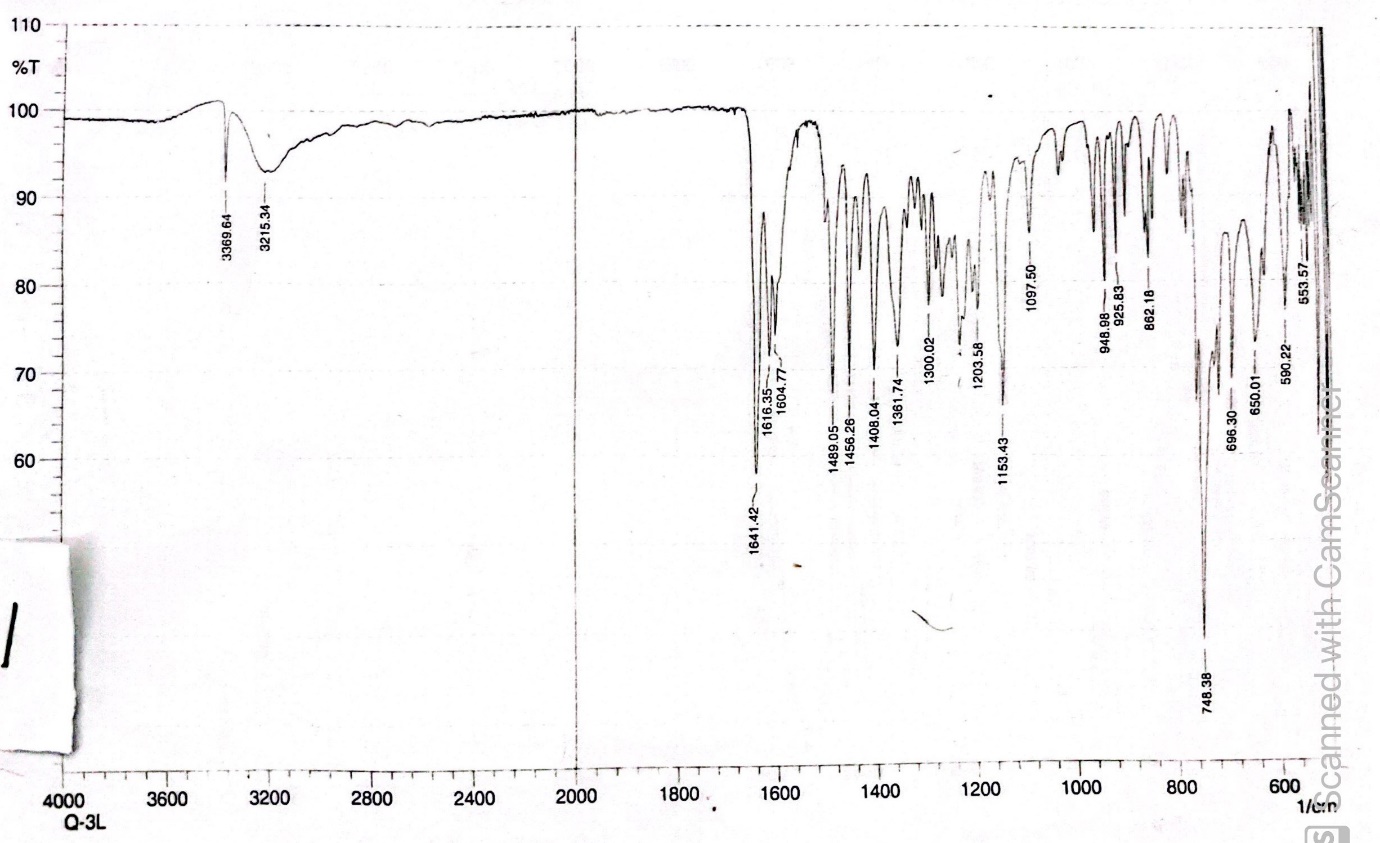
**

**Figure 4S: IR spectrum of H-AHMB**

**
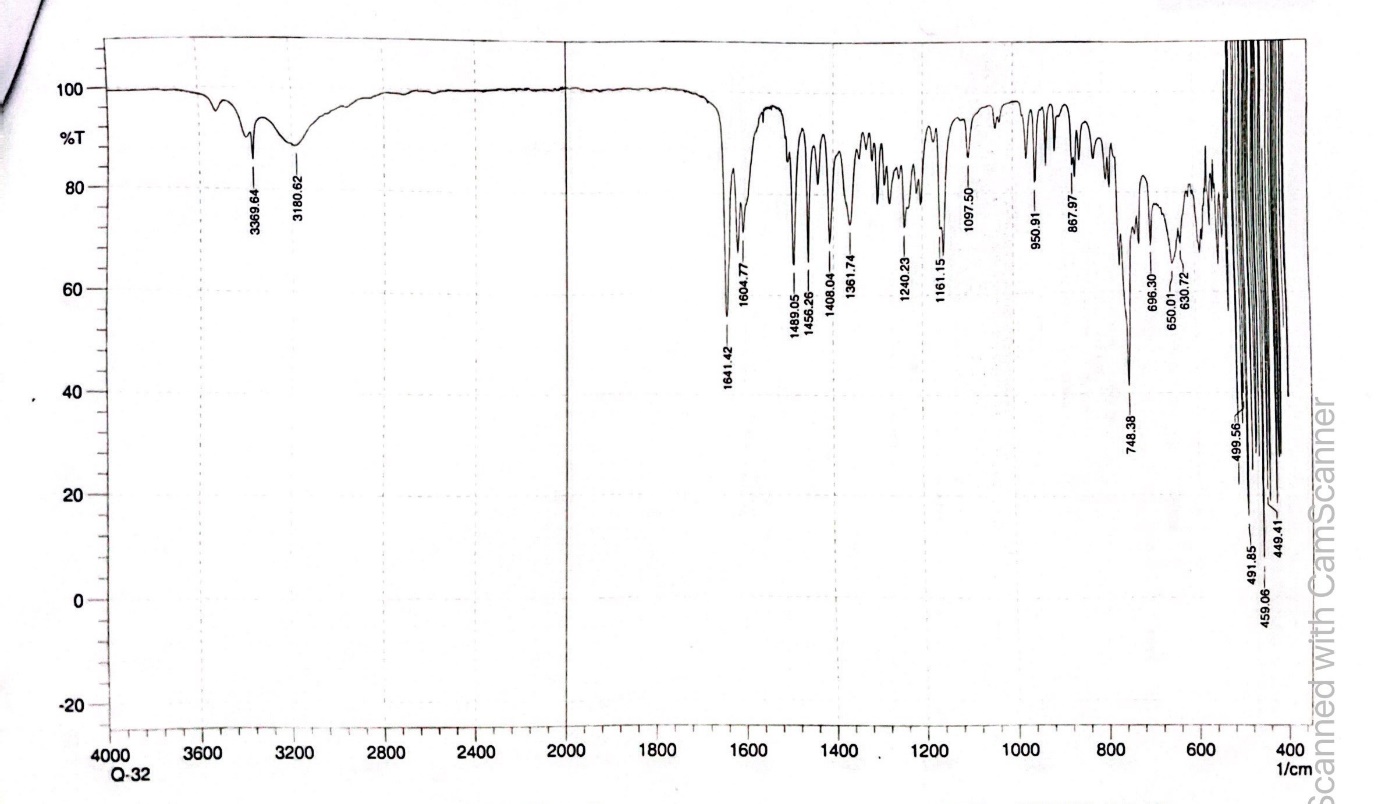
**

**Figure 5S: IR spectrum of Co-AHMB**

**
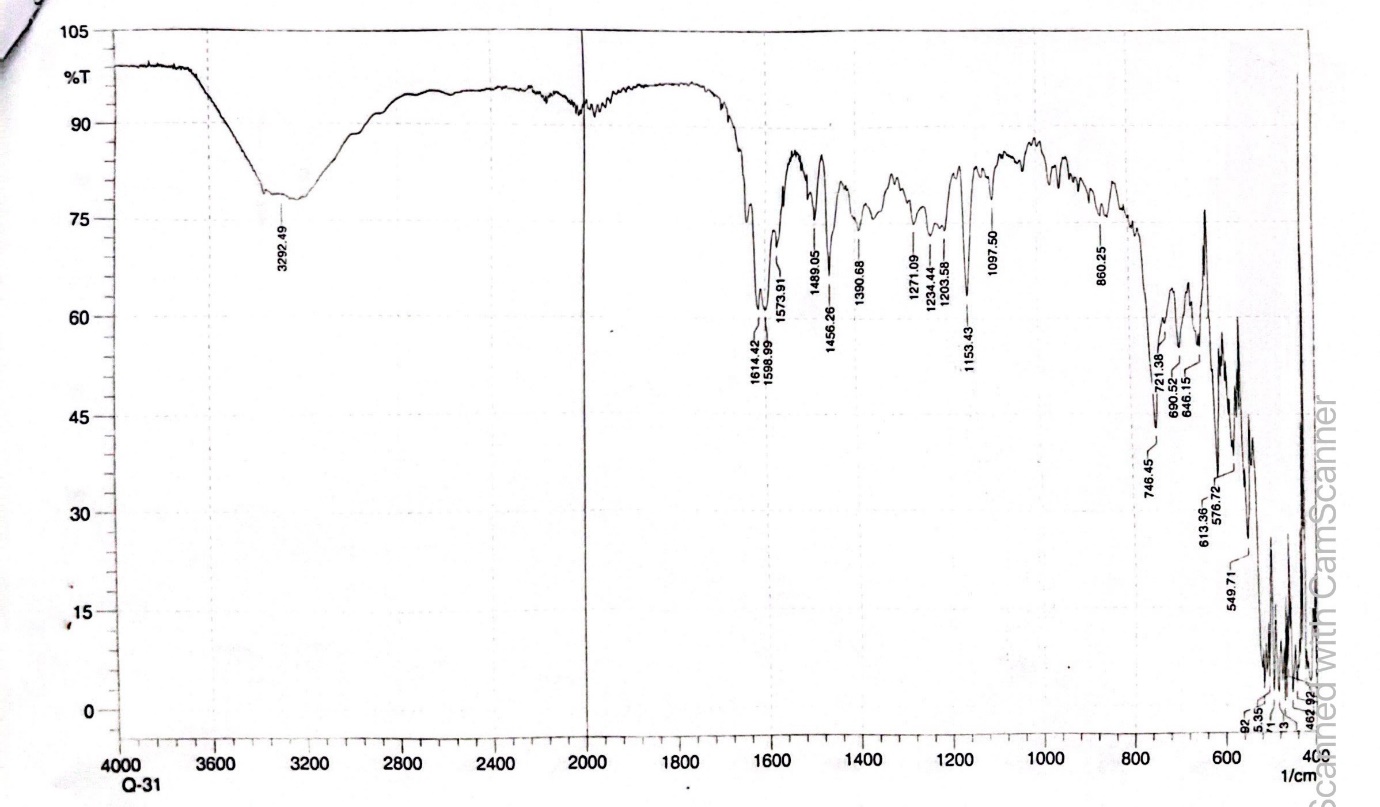
**

**Figure 6S: IR spectrum of Ni-AHMB**

**
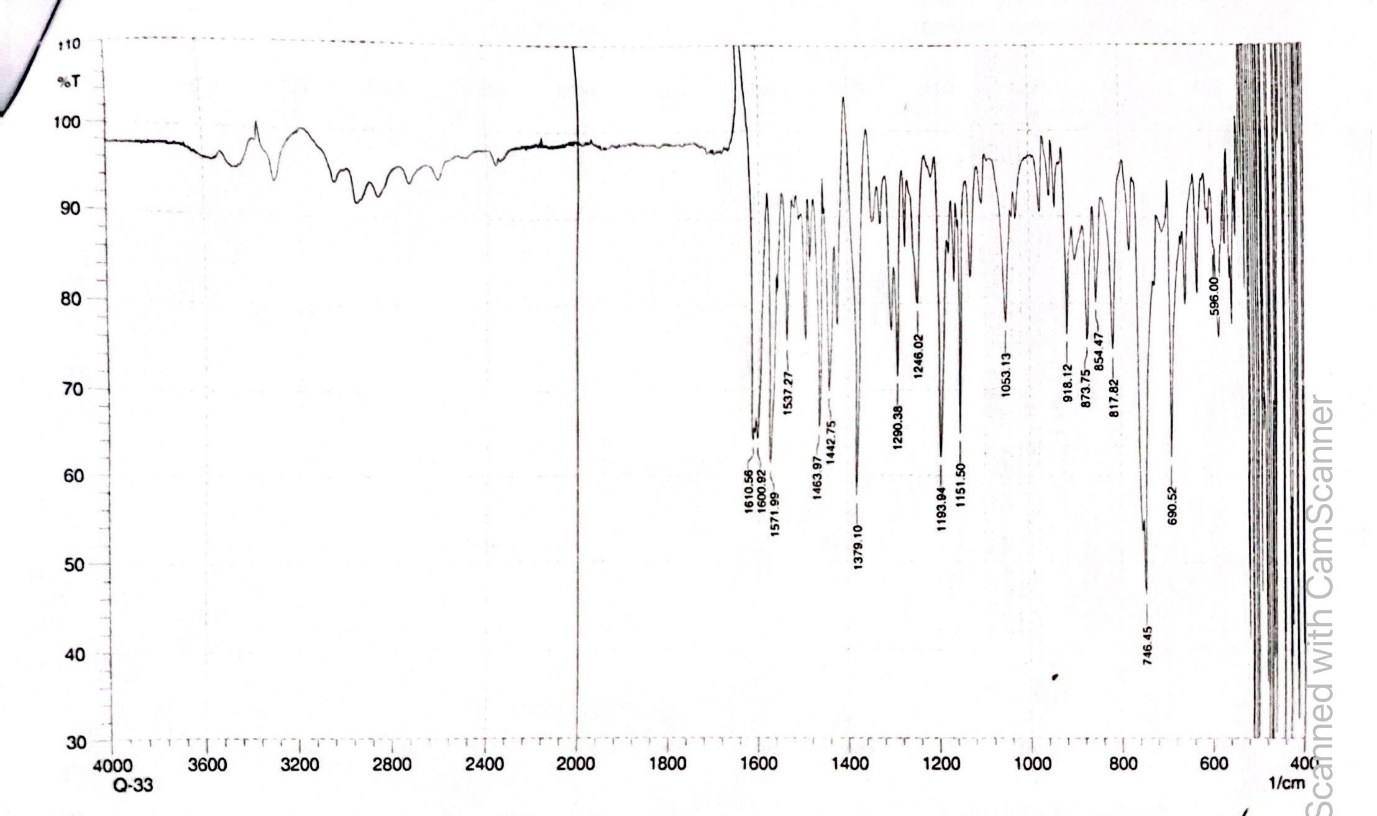
**

**Figure 7S: IR spectrum of Cu-AHMB**

**
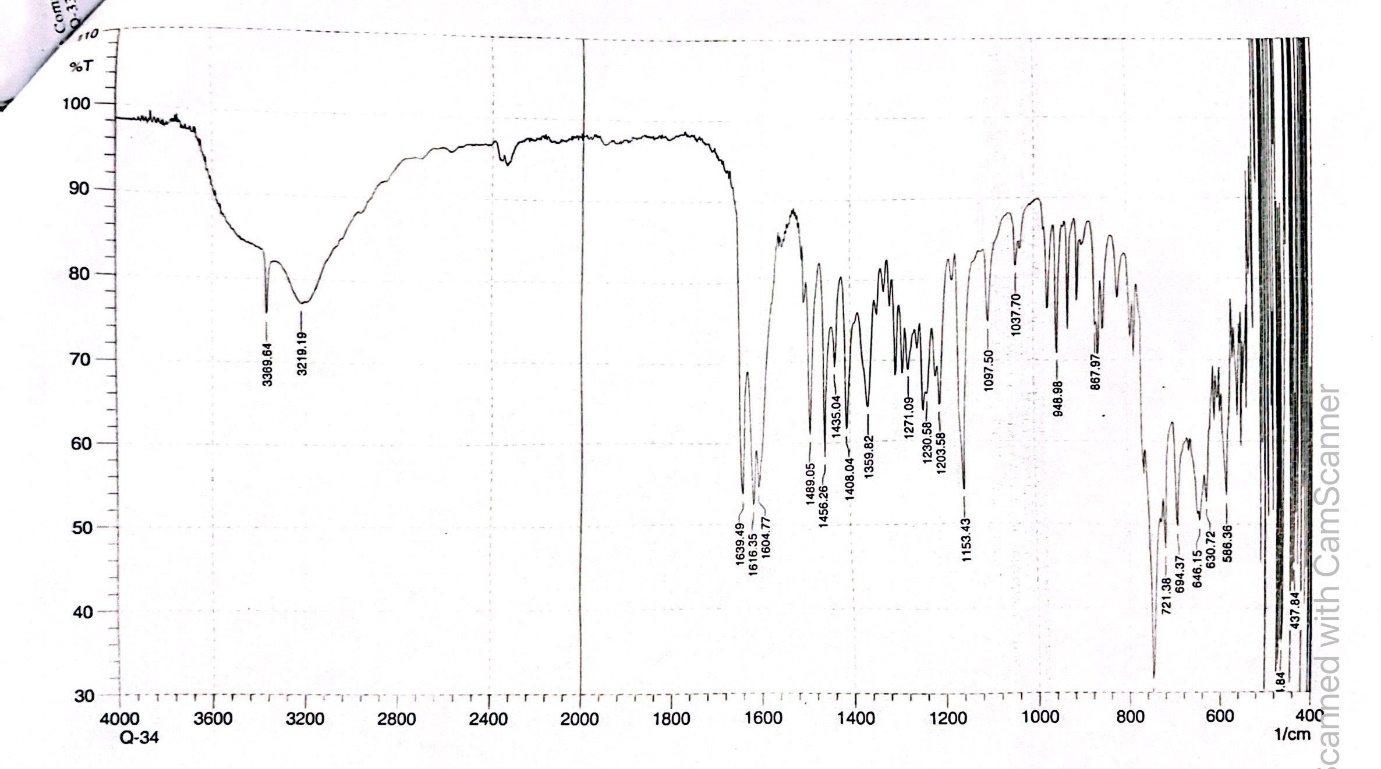
**

**Figure 8S: IR spectrum of Zn-AHMB**
